# Evolutionary divergence of the *ABO* and *GBGT1* genes specifying the ABO and FORS blood group systems through chromosomal rearrangements

**DOI:** 10.1038/s41598-017-09765-2

**Published:** 2017-08-24

**Authors:** Fumiichiro Yamamoto

**Affiliations:** 1 Laboratory of Immunohematology and Glycobiology, Josep Carreras Leukaemia Research Institute (IJC), Campus Can Ruti, Badalona, Barcelona Spain; 2 Programa de Medicina Predictiva i Personalitzada del Càncer (PMPPC), Institut d’Investigació Germans Trias i Pujol (IGTP), Campus Can Ruti, Badalona, Barcelona Spain

## Abstract

Human alleles at the *ABO* and *GBGT1* genetic loci specify glycosylation polymorphism of ABO and FORS blood group systems, respectively, and their allelic basis has been elucidated. These genes are also present in other species, but presence/absence, as well as functionality/non-functionality are species-dependent. Molecular mechanisms and forces that created this species divergence were unknown. Utilizing genomic information available from GenBank and Ensembl databases, gene order maps were constructed of a chromosomal region surrounding the *ABO* and *GBGT1* genes from a variety of vertebrate species. Both similarities and differences were observed in their chromosomal organization. Interestingly, the *ABO* and *GBGT1* genes were found located at the boundaries of chromosomal fragments that seem to have been inverted/translocated during species evolution. Genetic alterations, such as deletions and duplications, are prevalent at the ends of rearranged chromosomal fragments, which may partially explain the species-dependent divergence of those clinically important glycosyltransferase genes.

## Introduction

The ABO system is one of the most important blood group systems in transfusion medicine^1^. This polymorphic system, of glycosylation, is composed of A and B oligosaccharide antigens expressed on red blood cells (RBCs), and also some epithelial and endothelial cells, and anti-A and anti-B antibodies against those antigens in the sera of individuals who do not express the antigens. The FORS system is another system of RBC polymorphism expressing low-prevalence Forssman oligosaccharide antigen (FORS1).

Functional *A* and *B* alleles at the *ABO* genetic locus encode blood group A and B glycosyltransferases (A and B transferases) with distinct sugar specificities. A transferase transfers an *N*-acetyl-d-galactosamine to oligosaccharide acceptor substrate, H substance, to produce A antigen, whereas B transferase transfers a galactose to the same acceptor to produce B antigen. *O* allele-encoded proteins are enzymatically inactive and do not possess either of the transferase activities. Accordingly, H substance remains without further modifications in blood group O individuals. Molecular genetic basis of *ABO* was elucidated in 1990 when we cloned human *A*, *B*, and *O* allelic cDNAs and correlated their nucleotide sequences with A and B antigen expression^2^. *A* and *B* alleles encode proteins that differ by 4 amino acid residues, and these substitutions were shown to be responsible for different sugar specificities of A and B transferases^3^. The majority of *O* alleles are inactive due to a single nucleotide deletion^2^, although inactivating missense mutations were also found^4, 5^.

Forssman antigen has recently been associated with blood transfusion compatibility. It was known that RBCs from rare individuals exhibiting the phenotype named A_pae_ reacted strongly with *Helix pomatia* lectin, weakly with polyclonal anti-A antibodies, but not with monoclonal anti-A antibodies. Because of positive reactivity to polyclonal anti-A antibodies, A_pae_ was considered to be an A subgroup. However, chemical characterization of A_pae_ RBC glycolipids has identified Forssman glycolipid^6^. Forssman glycolipid synthase (FS: EC 2.4.1.88) encoded by *GBGT1* gene catalyzes the final step of Forssman antigen biosynthesis. Molecular genetic analysis demonstrated that A_pae_ individuals had a dominant-acting functional FS containing the Arg296Gln substitution when compared with that of ordinary non-A_pae_ individuals^6^. The International Society of Blood Transfusion (ISBT) has recognized FORS system as the 31st blood group system^6^.

*ABO* and *GBGT1* genes are evolutionarily related, belonging to the same α1–3 Gal(NAc) transferase gene family^7, 8^. Other members include *A3GALT2*, *GGTA1*, and *GLT6D1* genes, and the number and repertoire of the genes vary widely from species to species, indicating that the number of those genes has expanded and contracted by recurrent duplications and deletions during vertebrate evolution, following a birth-and-death evolution type. There are other species than humans that possess either one or both of the *ABO* and *GBGT1* genes. These species were initially identified immunologically. Expression of A/B antigens was examined in tissues of domestic and African wild animals^9^ and primates^10^. Studies on FORS1 antigen expression categorized vertebrates into Forssman antigen-positive and Forssman antigen-negative species^11^. Molecular and functional analyses were later performed. The *ABO* genes were investigated of some animal species, including primates^12, 13^, mice^14^, pigs^15^, and rats^16, 17^. The *GBGT1* gene cDNA encoding a functional FS was initially cloned from a dog^18^. Human *GBGT1* gene-encoded FS protein was found to suffer from structural deficiency^19^. Through functional analyses of Forssman-positive mouse and Forssman-negative human FS chimeras and their *in vitro* amino acid substitution constructs, we have shown that human *GBGT1* gene from ordinary individuals contains 2 inactivating amino acid substitutions, Gly230Ser and Gln296Arg, when compared with the functional murine *GBGT1* gene^20^. In addition to humans, structural deficiencies of *GBGT1* genes were also characterized in chimpanzees, gorillas, macaques, and cattle. No equivalent gene was found in rats, rabbits, or *Xenopus tropicalis* frogs.

In spite of the fact that structural deficiencies of gene-encoded glycosyltransferases have been well elucidated for the *ABO* and *GBGT1* genes, molecular mechanisms/forces causing gene disappearance in some species were unknown. During the past decade genome sequences have been determined of a variety of species, thanks to the genome sequencing projects. Through the annotation efforts, *ABO* and *GBGT1* genes have been identified in the genomes of dozens of species^8^. Therefore, taking advantage of genomic information available from public gene/sequence databases, attempts have been made to decipher those molecular forces. Changes during vertebrate evolution of the chromosomal region encompassing the *ABO* and *GBGT1* genes have been investigated, as well as the regions encompassing other members of the α1–3 Gal(NAc) transferase family genes. Here, I propose the following theory; chromosomal rearrangements have played a significant role in the generation of complex species-dependent gene distribution, by causing duplications and deletions of those glycosyltransferase genes of critical importance in transfusion and transplantation medicines.

## Results

### Human 9q34.13-ter chromosomal region and corresponding regions from other vertebrate species manifest both similarities and differences

The list of species analyzed in detail is shown in Table 1. There are 88 species consisting of 2 reptiles, 24 birds, and 62 mammals. They are numbered from 1 to 88 based on phylogenetic distance. In addition to common and scientific names, Class, Order, and Family (Infraclass and Infraorder if any) names, annotation versions, gene assembly versions, and numbers of contig gaps are also shown in Supplementary Table 1.

**Table 1:** The list of species analyzed in this study.

**Mammalia**	
*Haplorrhini*	1. Human, 2. Chimpanzee 3. Pygmy chimpanzee, 4. Western gorilla, 5. Sumatran orangutan, 6. Northern white-cheeked gibbon, 7. Rhesus macaque, 8. Crab-eating macaque, 9. Olive baboon, 10. Green monkey, 11. Golden snub-nosed monkey, 12. White-tufted-ear marmoset, 13. Bolivian squirrel monkey
*Strepsirrhini*	14. Small-eared galago
*Scandentia*	15. Chinese tree shrew
*Lagomorpha*	16. American pika
*Rodentia*	17. Thirteen-lined ground squirrel, 18. Long-tailed chinchilla, 19. Lesser Egyptian jerboa, 20. Prairie vole, 21. Chinese hamster, 22. Golden hamster, 23. Prairie deer mouse, 24. Laboratory mouse, 25. Rat, 26. Upper Galilee mountains blind mole rat, 27. Naked mole-rat, 28. Damara mole-rat, 29. Domestic guinea pig, 30. Degu
*Cetartiodactyla*	31. Alpaca, 32. Bactrian camel, 33. Chiru, 34. Sheep, 35. Goat, 36. Cattle, 37. River buffalo, 38. Yangtze River dolphin, 39. Sperm whale, 40. Killer whale
*Carnivora*	41. Cat, 42. Dog, 43. Ferret, 44. Polar bear, 45. Pacific walrus
*Perissodactyla*	46. Horse, 47. Southern white rhinoceros
*Chiroptera*	48. Brandt’s bat, 49. David’s myotis, 50. Big brown bat, 51. Black flying fox, 52. Large flying fox
*Eulipotyphla*	53. European shrew, 54. Star-nosed mole, 55. Western European hedgehog
*Afrotheria*	56. Cape golden mole, 57. Small Madagascar hedgehog, 58. Cape elephant shrew, 59. Aardvark, 60. Florida manatee
*Metatheria*	61. Opossum, 62. Tasmanian devil
**Aves**	
	63. Saker falcon, 64. Peregrine falcon, 65. Budgerigar, 66. Collared flycatcher, 67. White-throated sparrow, 68. Medium ground-finch, 69. Zebra finch, 70. Tibetan ground-tit, 71. Common canary, 72. American crow, 73. Hooded crow, 74. Downy woodpecker, 75. Golden eagle, 76. Bald eagle, 77. Crested ibis, 78. Emperor penguin, 79. Adelie penguin, 80. Killdeer, 81. Chimney swift, 82. Common cuckoo, 83. Rock pigeon, 84. Chicken, 85. Turkey, 86. Ostrich
**Reptilia**	
*Crocodylia*	87. Chinese alligator
*Testudines*	88. Green sea turtle

A total of 88 species were analyzed. They were numbered as shown. Detailed information may be found in Supplementary Table 1.

**Figure 1:**
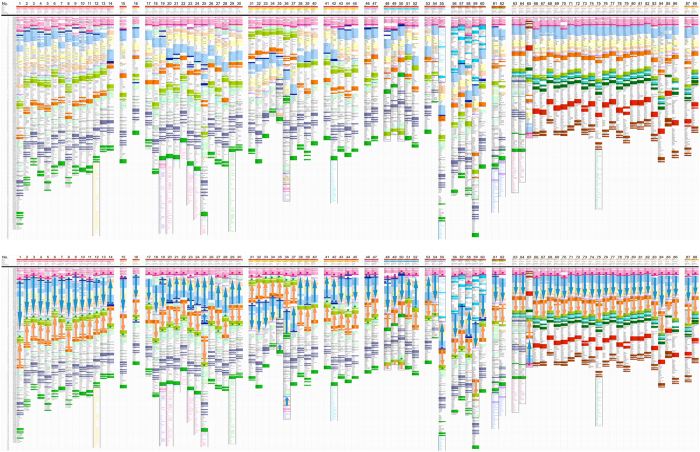
Schematic gene organization of human chromosome 9q34.13-ter and corresponding regions from other vertebrate species. Top panel: Gene maps of the chromosomal regions corresponding to human chromosome 9q34.13 to qter. The qter regions are dissimilar between reptiles/birds (except falcons) and mammals. Therefore, genes in that region are not equivalent. Additionally, there are species whose qter is fused with another chromosome or its fragment. Genes in that region are typed in a different color. Conspicuous differences are marked with black line boundaries. Species are aligned more or less according to the phylogenetic distance with respect to humans (sp. 1) and green sea turtle (sp. 88), although their placement may not be free of errors. Clusters of genes are color-coded to facilitate the identification of corresponding regions. The *ABO* and *GBGT1* genes, including partial genes, are shown in dark blue and pink, respectively. Bottom panel: Differences in the orientation of selected chromosomal fragments. The blue and orange arrows show the chromosomal fragments that span from *ABO* gene to *MRPS2* gene and the fragments that span from *FAM69B* to *KCNT1* genes, respectively. The asterisks (*) in dark blue, pink, and green color indicate *ABO*, *GBGT1*, and *GLT6D1* genes, respectively.

Genes in a chromosomal region from the *AK8* gene to qter were mapped, and are shown schematically from top (*AK8*) to bottom (qter) in columns in the top panel of Fig. 1. The original worksheet containing all the data used to prepare this figure is found in Supplementary Table 2. Gaps breaking chromosomal continuity are marked by a symbol (///). In order to facilitate the identification of corresponding segments, several genes of a cluster are coded (highlighted) in a color. When the qter is physically linked to another chromosome or its fragment, those genes are also included, at least partially if the gene list is long. Human chromosome is shown at the leftmost column (species 1), and the green sea turtle chromosome is shown at the rightmost column (sp. 88). The other species are more or less aligned based on phylogenetic relationship except within an Order. This way, progressive changes in evolution may be outlined.

Extensive similarities are observed in the kinds, numbers, and orders of genes, as well as their chromosomal locations. However, numerous differences are also identified. Several eminent examples are indicated with black lines surrounding the chromosomal fragments exhibiting differences. In some species the chromosomal end is fused with another chromosome. Several rodent species (sp. 20–25) share the joining partner, indicating that the speciation occurred after, and not before, the chromosomal end fusion. Shared joint partners are also observed in Metatheria and falcons in Aves. In other species, joining partners are unique (sp. 12, 19, 29, 42, 55, and 75). An insertion of chromosomal fragment (*FXN* – *PIP5K1B*) is seen in Afrotheria species (sp. 56–60). The portion of the insert is also found in Western European hedgehog (sp. 55). A paracentric inversion involving qter is observable in 3 bat species (sp. 48–50). Because it is not present in flying foxes (sp. 51 and 52), the inversion seems to have occurred after the separation of those two groups in Chiroptera. The q-ter side of chromosome may have been translocated to another location in Aardvark (sp. 59). Additional inversions are found in cattle (sp. 36) and budgerigar (sp. 65) among others.

It is noteworthy that Saker and Peregrine falcons (sp. 63 and 64) have a qter side of the chromosome distinct from other bird species. Surprisingly, that chromosomal fragment is almost identical with mammalian species (sp. 1–62) except for a small segment containing two dozens genes (*DPP7* – *RABL6*) in the opposite orientation and the qter joining. Chicken, turkey, and other birds share the same orientation of that segment, suggesting that a change in the direction occurred after the separation of mammals (marsupials) from birds. Monotreme platypus (*Ornithorhynchus anatinus*) has the chromosomal end more homologous to other birds than falcons (data not shown). Therefore, it seems that the translocated chromosome was inherited in a trans-species manner, bypassing monotremes.

### *ABO* and *GBGT1* genes are located at the ends of rearranged chromosomal fragments

The bottom panel of Fig. 1 is a modified version of the top panel. Blue arrows indicate the chromosomal fragments spanning from the *ABO* gene to the *MRPS2* gene, marking their origin and terminus, respectively, irrespective of the actual presence/absence of those genes so far as the region is homologous. Similarly, orange color arrows indicate the chromosomal fragments from *FAM69B* to *KCNT1*. Obviously, there are several different combinations of arrow locations and orientations, suggesting that chromosomal rearrangements such as inversions and translocations happened more than once during the evolution.

As in the top panel, the *ABO* and *GBGT1* genes are color-coded in dark blue and pink, respectively. However, in the bottom panel they are also marked with an asterisk (*) in dark blue (*ABO*) or in pink (*GBGT1*), which makes it easier to recognize that there are species with and without *GBGT1*. There are also species with and without *ABO*. Furthermore, there are species having multiple *ABO* genes including partial and non-functional genes (sp. 14, 19, 20, 21, 23, 25, 42, 44, 46, 48, 50, 58, and 60). Contrastingly, only 1 or none *GBGT1* gene exists per each species excluding fish species where more than 1 *GBGT1* gene may be present (2 for Playfish, 3 for Sticklebeck, and 10 for Zebrafish, for instance). In addition to the variation in gene number, differences are also observed in the gene locations. Surprisingly, *GBGT1* and *ABO* were found located at, or close to, boundaries of the rearranged chromosomal fragments.

### Genetic gain and loss are frequent of the *ABO* gene

In order to examine the relationship between the gene location being at the boundary of translocated/inverted chromosomal fragment and the frequency of genetic gains/losses, gene number was counted of 25 genes from the chromosomal region analyzed. Those genes are: *AK8*, *CEL*, and *RALGDS* (highlighted in rose color); *GBGT1* (pink); *ABO* (dark blue); *SURF6*, *MED22*, *SURF4*, and *ADAMTS13* (pale blue); *DBH*, *VAV2*, and *WDR5* (light yellow); *COL5A1* and *OLFM1* (tan); *MRPS2* (lavender); *KCNT1*, *CAMSAP1*, and *UBAC1* (lime); *CARD9*, *SNAPC4*, *PMPCA*, *INPP5E*, and *SEC* *16* *A* (white); and *NOTCH1* and *FAM69B* (orange). Excluding *ABO*, they were selected from genes common between human and chicken species from different sub-chromosomal portions of the region as manifested by the same colors highlighted in Fig. 1, and their presence/absence in other species has been investigated. In addition to the *ABO* and *GBGT1* gene, *MRPS2*, *KCNT1*, and *FAM69B* are also located at or close to the boundaries of rearranged chromosomal fragments.

**Figure 2:**
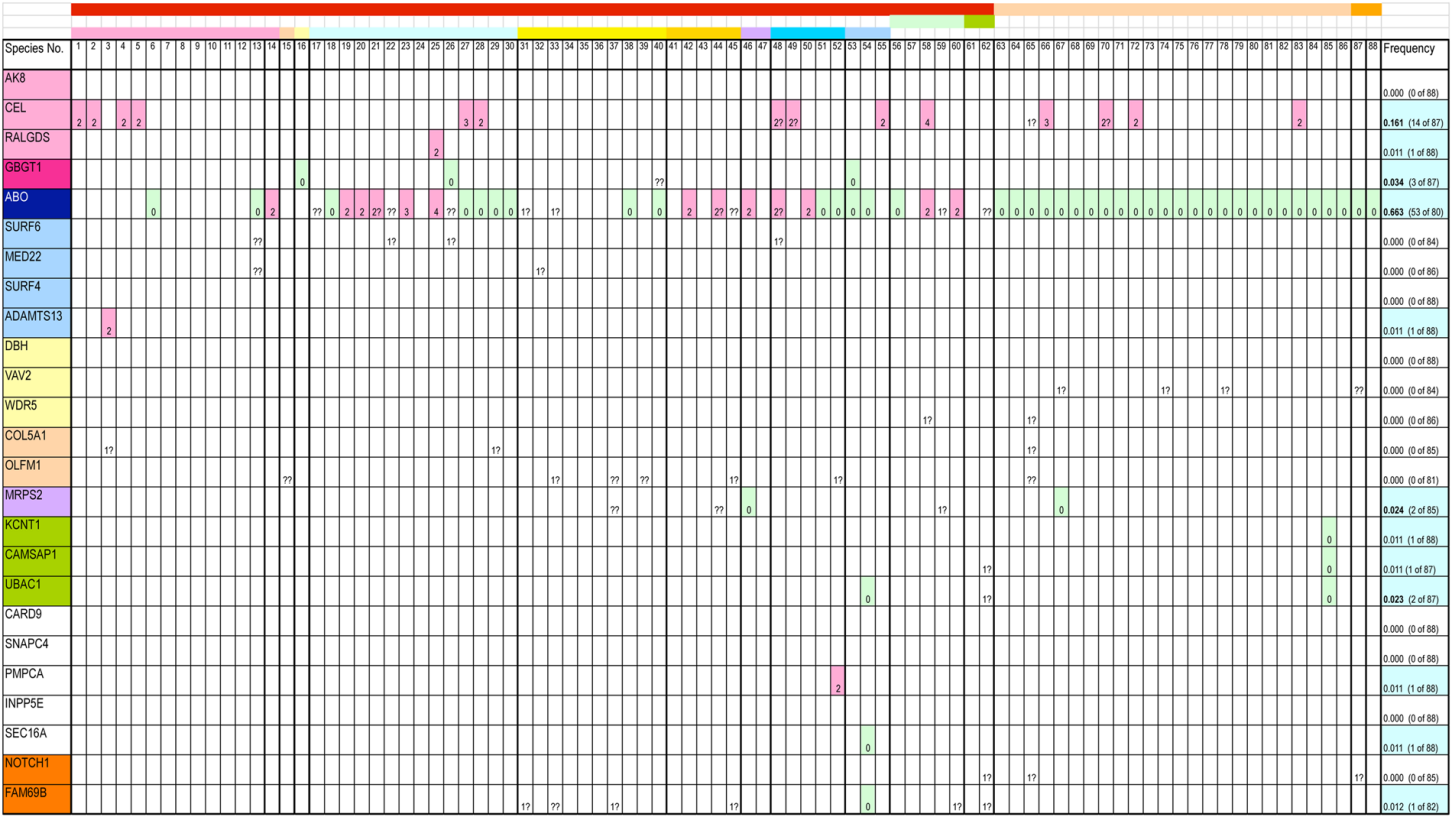
Gene copy number variations. Gene copy numbers were investigated of 25 genes from 88 vertebrate species. Please refer to Table 1 and Supplementary Table 1 for details of the species analyzed (sp. 1–88). The names of the 25 genes analyzed are shown in the leftmost column. They are also highlighted in the same colors as in Fig. 1 and Supplementary Table 2. Only the deviations from one copy are shown in this table. In other words, the open cells indicate that the copy number is one. The numbers 0, 2, 3, and 4 mean zero, two, three, and four copies, respectively. In order to facilitate visualization, genetic gains and losses are also highlighted in rose and light turquoise colors, respectively. Contig disruption is shown by question marks, with (??) indicating that the gene(s) are likely located in a contig gap, and (1?) and (2?) suggesting that at least one and two genes are present. Due to contig discontinuity, the exact copy number was not determined of those cases. The frequency of gene gain/loss was calculated for individual genes by dividing the number of species exhibiting genetic gain or loss by the number of species whose copy number was determined, and the results are shown in the rightmost column.

Data in Supplementary Table 2 were applied to quantification. The gene copy numbers were counted inside the chromosomal region. Results without phylogenetic consideration are shown in Fig. 2. The numbers indicate the copy numbers of full and partial genes combined, and only the deviations from one copy number are shown. The (??) marks show that the gene(s) are likely located in a contig gap, and therefore, the gene number was not determined. The (1?) and (2?) marks, respectively, indicate the presence of at least 1 and 2 genes, however, the actual number was not determined because the continuation was disrupted. DNA fragment containing *KCNT1*, *CAMSAP1*, and *UBAC1* genes seems to have translocated to another location in turkey (sp. 85). It should be noted that data used for analysis were not complete, and may have contained errors. They listed only the single genome for most species and lacked the information on polymorphism although the *ABO* gene copy number may vary as shown in rats and pigs^9, 15^. Genetic gain/loss frequency was calculated by dividing the number of species exhibiting genetic gain or loss by the number of species whose copy number was determined. The frequency proved to be high (0.663) for the *ABO* gene. A total of 53 out of 80 species showed a change in the gene number. Those 53 species are divided into 13 exhibiting genetic gain and 40 with genetic loss. Additionally, genetic loss was found of the *GBGT1* gene in 3 species, and genetic gain of *CEL* in 14 species. The *CEL* gene encodes carboxyl ester lipase involved in the hydrolysis and absorption of cholesterol and lipid-soluble vitamins. A recombined allele of the lipase gene *CEL* and its pseudogene *CELP* was shown to confer susceptibility to chronic pancreatitis^21^. The other 22 genes showed few copy number alterations.

**Figure 3:**
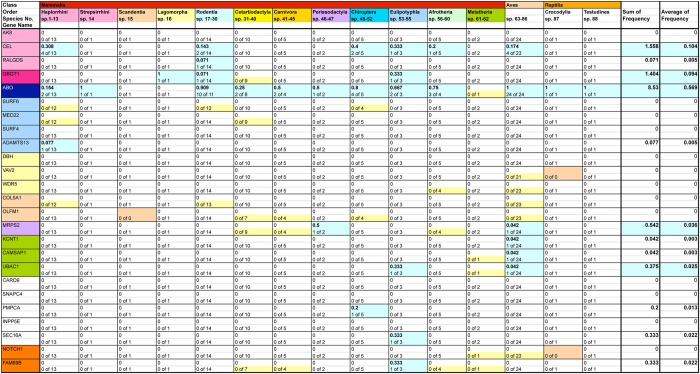
Frequencies of genetic gains/losses with considering phylogeny. In order to reduce the unbalancing effects due to disproportional numbers of species analyzed, information on the historical relationships of lineages was introduced. The same 25 genes that were analyzed without considering phylogeny were also analyzed. The 88 species were divided into 15 separated groups based on a phylogenetic tree of vertebrate species as shown in Table 1. The top 2 rows of the table show the Class and Order. Birds (sp. 63–86) were gathered into a single group. Frequencies of genetic gains/losses were calculated of each gene in evolutionarily related species in a group, and they are shown of individual groups in the single columns. Positive values are highlighted in light turquoise color. When frequency values were unobtainable because no species were determined of genetic gain/loss, those “cells” are highlighted in tan color and they were excluded from average calculation. Those values from different groups were summed up for individual genes, and the totals and averages are shown in the two rightmost columns.

The same data were further analyzed, taking into account the evolutionary relationships between species. For this purpose, genetic gain/loss frequency was calculated of species within the same taxonomical group, and the sum and average were obtained for individual genes. Results are shown in Fig. 3. The inclusion of phylogenetic consideration into quantification changed the frequency values, however, did not alter the conclusion that genetic gain and loss are frequent with the *ABO* gene.

### Expansion and transposition of *LCN1/3/4* genes may have promoted the *ABO/GBGT1/GLT6D1* gene evolution

Lipocalins (LCNs) are a family of proteins with varied biological functions including the transport of small hydrophobic molecules such as steroids, bilins, retinoids, and lipids^22, 23^. The members of this family share relatively low sequence homology but exon/intron structure and three-dimensional protein folding are highly conserved. The *LCN1/3/4* genes denoting *LCN1*, *LCN3*, *LCN4*, *OBP2A*, *OBP2B*, *PAEP*, *VEGP1*, and *VEGP2* genes seem to have emerged, possibly from *LCN9 or LCN15* in marsupials and duplicated/expanded as chromosomal recombination changed their locations/directions through species evolution. As a result, many lipocalin genes are clustered on the q-arm of chromosome 9.

The *GLT6D1* (glycosyltransferase 6 domain containing 1) gene is evolutionarily related to *ABO* and *GBGT1* ^8^. Whether *GLT6D1* encodes a functional glycosyltransferase or not remains to be determined, however, a genome-wide association study (GWAS) has associated polymorphic markers of single nucleotide polymorphism (SNP) at this genetic locus to the periodontitis susceptibility^24^. In the bottom panel of Fig. 1 the *GLT6D1* genes are marked with a green asterisk. *GLT6D1* genes are present at the ends of orange arrows in Afrotheria, surrounded by *LCN9* and *LCN1/3/4*.

**Figure 4:**
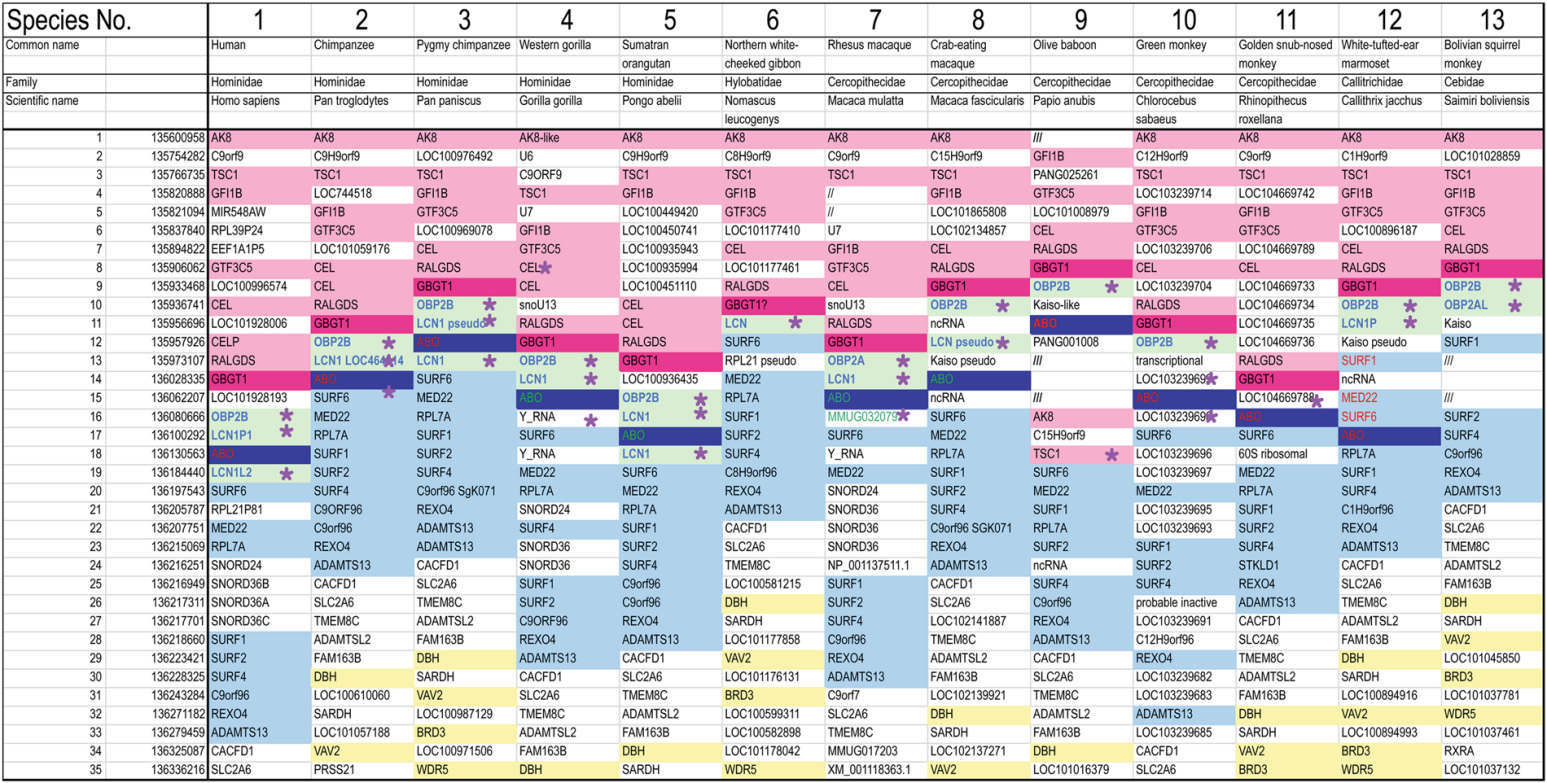
*LCN1/3/4* gene distribution around the *GBGT1* and *ABO* genes in primates. Genes in the chromosomal region in the vicinity of *ABO* and *GBGT1* in primates are shown. The *ABO* and *GBGT1* genes are shown in dark blue and pink, respectively. The *LCN1/3/4* genes annotated and homologous sequences detected by the BLAST search are indicated with purple asterisks. In order to fit into a cell, “ENS” and “00000” was removed from the ENS number names, for instance, PANG025261 for ENSPANG00000025261.

*LCN1/3/4* genes are mostly clustered in the boundaries between *GBGT1* and *ABO*, *MRPS2* and *GLT6D1*, *GBGT1* and *MRPS2*, *GBGT1* and *GLT6D1*, *ABO* and *GLT6D1*, *ABO* and *FAM69B*, and between *GLT6D1* and other *LCN* genes such as *LCN6/8/10/15*. The chromosomal regions around the *ABO* and *GBGT1* genes in primates were enlarged and are shown in Fig. 4. *Otolemur garnettii* (small-eared galago) belonging to the Infraorder of Strepsirrhini is not included because its chromosomal organization (sp. 14) is different from other primates (Haplorrhini) (sp. 1–13). In addition to the annotated *LCN1/3/4* genes, additional homologous sequences were identified close to the *ABO* genes by BLAST search. They are both indicated with a purple asterisk. The *LCN1/3/4* genes and their non-functional pseudogenes were found flanking the *ABO* gene on both sides in many primate species.

### Chromosomal fragmental rearrangements have been uncommon during mammalian evolution around the external boundaries of major histocompatibility gene complex locus

The chromosomal organization and its boundaries have also been analyzed of another highly polymorphic locus, the major histocompatibility complex (MHC), which plays a key role in the adaptive response to pathogens^25^. The human MHC locus, also known as HLA (human leukocyte antigen) locus, spans over a 3.6-megabase (3.6 Mb) stretch within chromosome 6p21, and contain many genes. The gene number and repertoire vary among species^26^. The analysis has been focused on their external boundaries in mammalian species (sp. 1–62). The gene order was found to be conserved with the proximal external boundary, from pter side, of *GABBR1*, *MOG*, *ZFP57*, ***HLA-F***, ***HLA-V***, ***HLA-G***, and ***HLA-A*** genes and the distal external boundary of ***HLA-DOA***, ***HLA-DPA1***, ***HLA-DPB1***, *COL11A2*, *RXRB*, *SLC39A7*, *HSD17B8*, and *RING1* genes. Among the 45 species whose gene orders were determined, the only exceptions were ferret (sp. 43) and polar bear (sp. 44), which exhibited the inversion of a small chromosomal fragment around the external distal boundary of the MHC Class I genes. In summary, the external boundaries of the MHC gene complex seem to have been relatively stable over the evolution of mammals.

## Discussion

It should be mentioned that genomic data used for this analysis were neither complete nor free from mistakes. Sequences and annotations are constantly being updated. As the number of species analyzed increases, identifying common patterns becomes easier although high number of species exhibiting the same pattern does not implicate that the pattern is prototypical. Also, as the number of individual animals analyzed increases, polymorphism may be found although individuals carrying such drastic deviations that cause infertility may have been eradicated from the species population^27^. A longer gene list does not always imply more genes, either, because the annotation level may be different and additional genes may later be annotated. Analyzing individual chromosomal maps gives a static view of the gene evolution. However, parallel analysis of numerous species in the context of phylogeny may allow more dynamic insights upon what occurred in the chromosomal organization during the evolution and when they happened.

The gene order analysis of chromosomal region surrounding the *ABO* and *GBGT1* genes in various species has demonstrated that those 2 genes are located at the edges of chromosomal fragments that have been rearranged multiple times in species evolution (Fig. 1). Quantitative analysis of gene copy number was performed of the 25 genes, including *ABO*, located in that chromosomal region. As shown in Fig. 2, genetic gains/losses have occurred frequently of the *ABO* gene. The *GBGT* gene seems to be missing in several species. Genetic gains/losses are rare of other genes, with the exception of frequent duplications of the *CEL* gene, which may have conferred some survival advantage. Similar results would likely be obtained if the analysis were expanded to include additional 25–35 genes that remain to be analyzed in the chromosomal region common between human and chicken species. The calculation of accurate frequency was difficult due to incomplete genomic data with contig gaps and erroneous annotations. It should also be noted that data used for quantification of genetic divergence between species listed a single representative genome of each species, and the gene absence in a genome deposited in genome database does not imply that all the individuals lack the gene.

Quantification was also performed, taking into account the phylogenetic relationships among species. This was necessary so as not to count the same event multiple times when it has been inherited by multiple species. For instance, the absence of the *ABO* gene in 24 species of birds does not imply the independent occurrence of genetic loss 24 times. Figure 3 shows the results obtained by re-calculating the genetic gain/loss frequency of species belonging to the same taxonomical group and summing up and averaging the frequencies for individual genes. Because several groups contained species with differential patterns of chromosomal fragmental orientations as shown in Fig. 1, those values may not be entirely accurate. However, the discordance effects due to different numbers of analyzed species may have been diminished to a certain extent.

We have previously shown that a prototype of α1–3 Gal(NAc) transferase family of genes is present in lampreys and that their functional genes are present in many vertebrate species^8^. In fishes the *A3GALT2* genes are located in the chromosomal locations (*GUCA1B* or *MAPK8IP1*, ***A3GALT2***, *LRP4*, *NELL1*) and (*FAM83E*, *EMP3*, ***A3GALT2***, *ZNF362*, *TRIM62*). In mammals they are located in a common location (*ZSCAN20*, *PHC2*, ***A3GALT2***, *ZNF362*, *TRIM62*). The fish *A3GALT2* genes in the latter category and the mammalian genes share on one side a similar gene set (***A3GALT2***, *ZNF362*, *TRIM62*), suggesting that a chromosomal translocation took place at the *A3GALT2* boundary during the transition from fishes to mammalian species. In fishes *GBGT1* genes are located in various locations, however, in amphibians, reptiles, birds, and mammals the *GBGT1* genes are linked, on one side, to the same set of genes (***GBGT1***, *RALGDS*, *CEL*, *GTF3C5*, *GFI1B*). Genes on the other side are diverse due to chromosomal rearrangements as shown in Fig. 1. The *ABO* genes are present in amphibians. The *FUT1/FUT2/Sec* genes encoding α1–2-fucosyltransferases, which catalyze the last biosynthetic step of the H substance, the acceptor substrate for A and B transferases, are also present in amphibians. The *GGTA1* and *GLT6D1* genes are present in some mammals in (*TTLL11*, *DAB2IP*, ***GGTA1(−1)***, ***GGTA1(−2)***, ***GLT6D1(−1)***, *STOM*, *GSN*) and (*OBP2A*, *PAEP*, ***GLT6D1(−2)***, *LCN9*, *SOHLH1*, *KCNT1*). Combining this information with data obtained from the present analysis, it may be said that chromosomal rearrangements have diversified the evolution of not only *ABO* and *GBGT1* genes, but also other members of α1–3 Gal(NAc) transferase family genes.

Contrastingly, chromosomal fragmental rearrangements were scarce around the external boundaries of the highly polymorphic MHC locus. The results implicate that being situated close to rearranged chromosomal fragments is not required for gene diversity or polymorphism. However, the gene order analysis performed of MHC was limited to the external boundaries in mammals. Considering that MHC gene families are found in all vertebrates and that varied repertoire of member genes, great allelic diversity, and polymorphism among member genes have resulted from gene duplications, rearrangements may likely be found inside the complex.

**Figure 5:**
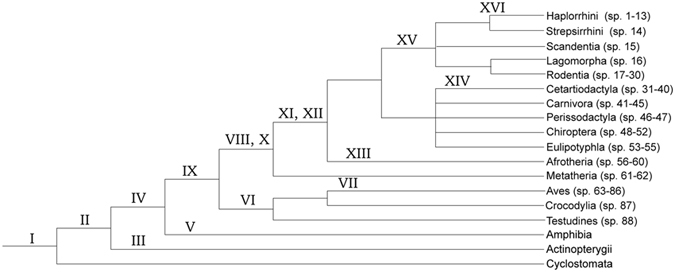
Major events taken place during the evolution of α1,3-Gal(NAc) transferase genes. Based on genomic information available and logical insights, 16 major events that might have occurred during the evolution of α1,3-Gal(NAc) transferase genes were deduced, and are schematically shown in a phylogenetic tree of vertebrate species. The events are numbered in Roman numerals from I to XVI. The species analyzed were categorized and are shown in number in Table 1. The explanations for individual incidents are presented in the Results section. It should be noted that those numbers might not accord with the order of occurrence during evolution.

A possible evolutionary scenario for α1–3 Gal(NAc) transferase genes involves at least sixteen major events (I-XVI) that might have occurred. They are marked in an approximate timing in a phylogenetic tree of vertebrate evolution in Fig. 5. The prototype of α1–3 Gal(NAc) transferase gene (similar to *A3GALT2*) appeared in lampreys (Event I). Functional *GBGT1* and *A3GALT2* genes appeared in fish by gene duplication and divergence (II). The *A3GALT2* gene was duplicated and they are present in two separate locations (III). In amphibians *ABO* genes appeared after the duplication of the *GBGT1* gene followed by divergence (IV). *Xenopus* frogs lost *GBGT1* due to chromosomal inversion (V). On the other hand, some reptiles and birds lost *ABO* (VI), and the chromosomal region has been relatively stable in those Classes. Falcon species (sp. 63–64) were separated from other bird species (sp. 65–86) by genomic alterations including the chromosomal translocation shown in Fig. 1 (VII). The translocated chromosome was somehow inherited to mammals (VIII). The *A3GALT2* genes were duplicated and one copy was inserted as the prototype of *GGTA1/GLT6D1* between the *DAB2IP* and *STOM* genes (IX). Another duplication occurred after the integration, and *GGTA1* and *GLT6D1* genes emerged in marsupials (X). In some mammalian species the *GGTA1* gene was further duplicated and two copies are present in tandem (XI). The *GLT6D1* gene was also duplicated, however, rather than remaining in tandem at the same location, one copy was transposed, together with the prototype of *LCN1/3/4* gene, to the terminal end of the chromosomal fragment indicated by the orange arrow in Afrotheria (sp. 56–60) in the bottom panel of Fig. 1, possibly accompanying with the chromosomal fragment rearrangement (XII). In this Infraclass, an additional chromosomal fragmental insertion also occurred (XIII). The *GLT6D1* and *LCN1/3/4* gene side of the fragment became physically linked to the *GBGT1* gene in Cetartiodactyla (sp. 31–40) excluding species in the Infraorder of Cetacea (sp. 38–40) (XIV).

In certain rodent and all the primate species, some *LCN1/3/4* genes remained at the boundary near the *GBGT1* gene even after the fragment translocation to another location or their copies were transposed there (XV). As shown in Fig. 4, the *LCN1/3/4* genes/pseudogenes flank the *ABO* gene on either side in many primates (XVI). This is intriguing, considering that Haplorrhini primates (New World Monkeys, Old World Monkeys, and Hominoids) exhibit monogenic ABO polymorphism as opposed to some vertebrate species having multiple non-allelic *ABO* genes. Because the evolution of α1–3 Gal(NAc) transferase family genes has been frequently associated with changes in chromosomal organization, the creation of *ABO* allelism in primates may not be an exception. I conjecture that unequal recombination that made both sides of the *ABO* gene flanked with the *LCN1/3/4* genes/pseudogenes may have contributed to the conversion of multigenic *A* and *B* genes in other species into monogenic *ABO* alleles in primates. Because the sequence motifs and *LCN* gene family could make assembly errors, further studies are needed to assess this hypothesis. In addition to *LCN* genes, surfeit genes, especially *SURF6* gene, are often found close to the *ABO* genes as they are indicated in blue in Figs 1 and 4. Although the long-term evolution of surfeit genes may be of scientific importance^28^, those genes are located inside the chromosomal fragment with the *ABO* gene at the end, and therefore, their involvement in recombination may be insignificant.

When we cloned human A transferase cDNAs in 1990, we identified CA dinucleotide repeats sequence in the 3′-UTR region of the human *ABO* gene^29^. We had a hard time to clone the cDNAs with a long 3′-UTR sequence possibly because of the CA repeats stretch. Therefore, it is not difficult to imagine that this sequence may be somewhat responsible for problematic nucleotide sequencing/contig alignment around the *ABO* gene in some species. Because the repeat region is located between the coding sequence (CDS) of *GBGT1* gene and the CDS of *ABO* gene, it might have caused chromosomal rearrangements at the junction. The possibility that observed rearrangements might have resulted from lower quality assemblies/annotations exists. However, the contig interruptions are not often within the junction, and the majority of interruptions are found in other chromosomal regions, suggesting that the rearrangements are real and not an artifact.

The finding of *ABO*, *GBGT1*, and *GLT6D1* genes at the boundaries of chromosomal fragments with previous history of rearrangements is meaningful. *A3GALT2* and *GGTA1* genes, other members of α1–3 Gal(NAc) transferase family, are not located at such instable boundaries. The external borders of the MHC Gene Complex are not, either. As shown in Fig. 2, the quantification of genetic gains/losses demonstrated differential frequencies among genes located at the ends of rearranged chromosomal fragments. The *ABO* gene showed the highest with 0.663, the *GBGT1* gene with 0.034, *MRPS2* with 0.024, and *KCNT1* with 0.011. It is evident that gene nature is critical in determining such differences, in addition to the gene position. Indispensable housekeeping genes had to be maintained even if chromosomal rearrangements took place at near-by locations. Accordingly, only the species without the genetic loss seem to have survived.

Chromosomal anomalies and gene copy number alterations are hallmarks of cancer^30^. The loss of tumor suppressor genes and the gain of oncogenes confer cancer cells with growth advantages. Accordingly, many genes may be lost and/or amplified during cancer evolution. However, species evolution has been more conservative. All the genetic information, which permits the survival of individuals, as well as the continuation of species over the generations, should be retained. Genetic gains may not necessarily be advantages, either, potentially disrupting physiological balances. As opposed to the genes in the chromosomal region that has been stable and unchanged over the evolution or the genes well within the rearranged chromosomal fragments, the genes at their borders are more likely to suffer from genetic alterations including losses and duplications. Actually, protein evolution was reported to be more than 2.2 times faster in chromosomes that had undergone structural rearrangements compared with co-linear chromosomes^31^. This enhancement in evolution may be eminent with genes dispensable for the individual’s survival. Residency of such polymorphic genes as *ABO* next to instable chromosomal structure prone to rearrangements may have provided an opportunity to further divergence. Furthermore, chromosomal fragmental inversions are known to accelerate speciation^32^. Therefore, the evolution of α1–3 Gal(NAc) transferase genes may not only have generated species-dependent divergence, but also have promoted speciation.

## Methods

### Retrieval of genomic information on the *ABO* and *GBGT1* genes and their surrounding genes in a variety of vertebrate species

In humans the *ABO* and *GBGT1* genes are located at band 34.13 on the q-arm of chromosome 9. Accordingly, we retrieved genomic information surrounding those two genes from the National Center for Biotechnology Information (NCBI) database, using Map Viewer (https://www.ncbi.nlm.nih.gov/mapview/). The information included gene annotation and description, gene order, gene orientation, the location of contigs and gaps. Human chromosomal region (132,725,574 bp – 138,394,717 bp) spanning from *AK8* gene on the 9q34.13 band to the end of chromosome (qter) was selected as the standard. Next, genomic annotations on genes in the corresponding chromosomal region were retrieved from other vertebrate species. When a gap exists between contigs, additional information was obtained from other databases including the Ensembl genome browser 86 (http://www.ensembl.org/index.html) in order to close or narrow down the gap.

### Selection of species for detailed chromosomal mapping

Fishes were excluded from further consideration because of rather incomplete chromosomal mapping and gene annotation. The chromosomal regions containing the homologous gene(s) were significantly different between fish species and also from other vertebrate species. In addition to fishes, genomic sequences and annotations were preliminary for many other species. Therefore, those species whose chromosomal organization was interrupted by more than 4 contig gaps within the selected region were also eliminated. A total of 88 species satisfied this criterion and were further analyzed (Table 1 and Supplementary Table 1).

### Manual lining-up of chromosomal regions

The retrieved gene orders from species without any contig gaps were first lined up in parallel with the standard human chromosomal organization in a Microsoft Excel table. Next, the species with a single gap were aligned. The determination of chromosomal fragment orientation was easy for those species because the two contigs were either proximal or distal to qter. Then, the species with 2 interruptions were added to the table. In those cases, the orientation of the middle fragment was deduced, following the configuration patterns of evolutionarily related species. This was possible because many of the disruptions occurred at different locations of chromosome. And finally, the species with 3 or 4 contig gaps were added to the table after lining-up the middle 2 or 3 fragments in the orientations with least contradictions with closely related species.

There were many genes with gene numbers, such as LOC… and ENS…, rather than gene names. In addition, orthologous genes have been occasionally named differentially in different groups of species, primates and rodents, for example. In an effort to make the annotation as accurate as possible, those genes were identified by annotation search in Gene Tree and also by BLAST using the nucleotide and protein sequences from one species as query sequences to search for homologous gene(s) in evolutionarily related species. Species were ordered according to phylogenetic distance. Clusters of genes were color-coded to identify homologous regions and differences in chromosomal organization. The *GBGT1* and *ABO* genes were identified and marked. In case that *ABO* or *GBGT1* genes were not annotated, the gene nucleotide sequences were retrieved from evolutionarily close species, and homologous sequences in the genome have been extensively searched, using BLAST. In some species the qter was found fused with another chromosome or its fragment. Those genes were typed in different colors. Supplementary Table 2 contains the information in an Excel file format.

The genes surrounding the external boundaries of the MHC locus were analyzed of mammals (sp. 1–62), using Ensembl Genome Brower and GenBank Species Genome Map Viewer, and they were ordered as described above.

### Quantification of genetic gains/losses with and without considering phylogeny

Data in Supplementary Table 2 were used for quantitative analysis of genetic gains/losses of 25 selected genes in the chromosomal region common among the species examined. The numbers of genes were counted of individual species and a table was prepared. The frequency of gene number alterations was calculated by dividing the number of species exhibiting changes by the number of species whose gene numbers were determined.

The same data from Supplementary Table 2 were also used to determine the genetic gains/losses, taking phylogeny into account. The number of species showing gene copy number alterations was counted separately for 15 different taxonomical groups shown in Table 1. The frequency was then calculated by dividing them by the number of species determined within a groups, and the values were summed up and also averaged for individual genes.

## Electronic supplementary material

Supplementary Table 1

Supplementary Table 2
